# UAV-Based Volumetric Measurements toward Radio Environment Map Construction and Analysis

**DOI:** 10.3390/s22249705

**Published:** 2022-12-11

**Authors:** Antoni Ivanov, Bilal Muhammad, Krasimir Tonchev, Albena Mihovska, Vladimir Poulkov

**Affiliations:** 1 Intelligent Communication Infrastructure Laboratory, Sofia Tech Park, 1784 Sofia, Bulgaria; 2 Faculty of Telecommunications, Technical University of Sofia, 1000 Sofia, Bulgaria; 3 Department of Business Development and Technology, Aarhus University, 7400 Aarhus, Denmark

**Keywords:** cognitive radio, radio environment maps, software-defined radio, spectrum utilization, unmanned aerial vehicles, volumetric measurements, wireless communications

## Abstract

Unmanned aerial vehicle (UAV)-empowered communications have gained significant attention in recent years due to the promise of agile coverage provision for a large number of various mobile nodes on the ground and in three-dimensional (3D) space. Consequently, there is a need for efficient spectrum utilization in these dense aerial networks, which is characterized through radio environment maps (REMs), the construction of which is an important research area. Nevertheless, due to the difficult collection of radio frequency (RF) data, there are limited works that are based on real-world measurement campaigns. This paper presents a novel experimental setup that includes a constellation of three UAVs, the communication signals of which are measured by a software-defined radio (SDR) mounted on a separate UAV. It follows a trajectory that defines the REM’s two-dimensional (2D) area on a plane, executed at four altitudes, to extend the REM to 3D. The measurements are then processed and their features (received mean power level, average difference of the mean power, percentage of meaningful correlations) are analyzed in the temporal, spatial, and frequency domains to determine the utilization of a 20 MHz band in the 2.4 GHz spectrum, as well as their variation with altitude. This analysis provides a base for research in reducing the amount of measurements (by identifying the regions of low and of high interest) and spectrum occupancy prediction for UAV-based communication coexistence.

## 1. Introduction

Recent developments in the Fifth Generation (5G) of wireless communications and beyond have shown increased heterogeneity in contemporary and future application scenarios (ultra-reliable low-latency communications, holographic telepresence, telemedicine, smart devices and sensors, military reconnaissance, emergency communications provision, augmented and virtual reality, remote robotic control) via diverse ground, aerial, marine, and space communication nodes with different physical and processing capabilities [1,2,3,4]. A prominent field of research is appointed to the unmanned aerial vehicle (UAV) design and their application for communication among UAVs, between UAVs and terrestrial nodes, and between UAVs and nonterrestrial (marine and space) nodes [3,5,6]. It considers the provision of services such as aerial BS for cellular users in emergency conditions or for terrestrial BS offloading, spectrum occupancy and network key performance indicators (KPIs) assessment for network planning and algorithm design, delivery of goods, intermediary data relay from space to terrestrial nodes, search and rescue, connectivity for rural and remote areas UAV-based backhaul, and environment monitoring [3,7,8]. Furthermore, flying ad hoc networks have gained more attention in the literature, being delay-tolerant and affording the possibility for relaying with satellite nodes with decreasing the overall latency in space–terrestrial communications [4]. Their implementation poses additional challenges in terms of handover, routing, three-dimensional (3D) radio propagation modeling, energy efficiency, and localization accuracy of the terrestrial nodes by the UAVs. As an example, a real-world UAV-based search and rescue platform was implemented and tested in [9]. It utilizes a modified YOLO neural network algorithm for objects recognition through the UAV’s onboard camera, and it also provides a secure connection with the ground-based user terminal. Further development for UAV path planning with physical threat detection in 3D environments was performed in [10,11]. Cubic spline interpolation for energy efficient path optimization and genetic algorithms are employed with significant improvement to the baselines. These services are dependent on the proper self-organization of the UAV communication nodes via appropriate resource allocation, user association, and UAV placement (also termed trajectory or path planning). To this end, asynchronous federated learning can be applied in the UAV relay nodes, with the added benefit of low computational complexity and increased privacy for hundreds of users [12]. Consequently, spectrum usage optimization is becoming an increasingly important aspect of the development of forthcoming communication networks, especially as it is expected that the number of UAVs will increase substantially in the near future [6]. Thus, the allocation of the limited frequency resources, whether in the licensed or unlicensed bands (UAV communications operating in both), and the identification/detection of any underutilized portions of them is a nontrivial problem, especially considering the UAVs’ dynamic movement in all three dimensions of space. A crucial step in designing the required algorithms is to determine the method by which the intelligent receiver is to estimate the radio environment parameters and adapt in accordance with their change in time, frequency, and space/location (i.e., volumetric spectrum analysis that includes spectrum sensing, channel quality assessment, and prediction) [13]. Integration of cognitive radio (CR) into UAVs for more efficient spectrum access has thus been established as a contemporary area of research with the aim of providing solutions to these demanding challenges [8,14]. Radio environment maps (REMs) have been established as one of the most prominent tools for the conception of dynamic spectrum access (DSA) methods [15]. They comprise a database of one or more radio environment parameters that are collected for a number of points that define a volume of space in a particular geographical indoor or outdoor area [16]. This study is focused on spectrum characterization through the received signal strength (RSS) and the parameters derived from it, and thus, according to the taxonomy introduced in [16], the type of REMs that are considered in this work are occupancy maps. The benefit of REMs for both static spectrum access and CR networks comes from the relaxation in terms of the processing complexity of spectrum analysis and resource allocation methods as they provide some a priori knowledge of the estimated parameters [15]. With their availability, both short- and long-term prediction of the spectrum occupancy and quality of service (QoS) parameters, as well as network planning, can be performed [16].

**Figure 1:**
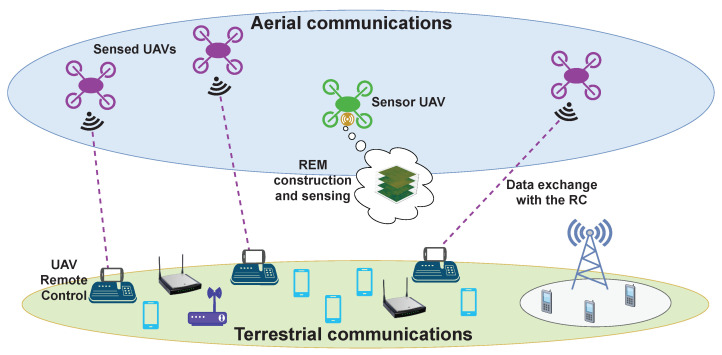
System model of the UAV-based sensor for aerial communications.

UAVs can, at the same time, utilize REMs for the optimization of their communications throughput (dependent on the flight path adaptation), and collect the measurements for the maps’ construction. For this reason, research in this area has recently attracted much attention by the scientific community [17,18,19,20,21,22,23,24,25,26]. REMs constructed via UAVs are usually estimated from limited number of measurements due to the difficulty of gathering the complete set of measurement samples that describes a large 3D volume due to the small flight time provided by the onboard batteries. Thus, when monitoring the radio environment, a UAV needs to determine an optimal trajectory that covers the regions of interest (RoIs) which provide the most meaningful data for the received signal distribution [23]. The purpose of an REM is determined by the geographical area (propagation conditions are affected by the number of obstructions and their physical characteristics), frequency band (aerial and space communications use diverse portions of the spectrum), and, consequently, by the application scenario for UAV communications (if such is strictly defined in a particular work) [6]. In addition, the coexistence of UAV communications and terrestrial cellular/unlicensed networks is a significant research area that includes CR- and REM-enabled spectrum sharing and resource allocation algorithms [6,27,28]. Moreover, characterizing the spectrum occupancy in 3D is also a crucial aspect in such studies as it includes the RSS fluctuations with altitude (a certain band may be underutilized at one altitude/height but heavily congested at another). Even though measurements and REM construction in a single altitude, i.e., on a two-dimensional (2D) plane as well as in a 3D volume, have both been explored in a substantial way in the literature, the amount of works that consider a UAV-based sensor for measurement collection in real time is limited [17,18,29]. Furthermore, most of the research examines frequency bands occupied by either a single individual or several terrestrial fixed transmitters of different communication standards [17,18,19,20,21,22,23,29,30,31,32]. This work considers the operation between the UAVs and their radio controllers (RCs) that uses the 2.4 GHz Industrial, Medical, and Scientific (ISM) bands and, more specifically, their occupancy in the presence of multiple active UAVs (Figure 1). The difference with recent relevant works (such as [20,21,30]) is the emphasis on real-world measurements of the signal features (extracted from the RF data) and their analysis, as well as the construction of volumetric REMs. These results can be a basis for spectrum occupancy prediction in the ISM bands to facilitate the coexistence of UAV-based communication nodes and licensed users. The experimental setup considers a “sensor UAV” that, thorough the onboard mounted software-defined radio (SDR), records the measured signal at a particular band following a predetermined trajectory at the same altitude. This set of measurements is performed for several different altitudes (planes) for the creation of a volumetric REM. The signals present in the spectrum are emitted by separate “sensed UAVs” as they communicate with their respective RCs. These UAVs form a constellation as they fly in a realistic manner in the same region in which the sensor UAV does. The focus of this work is not the REM construction itself, but the RF data analysis and its potential as a basis for spectrum occupancy prediction in UAV-based cognitive radio networks. The results can also be used as a benchmark for REM interpolation and estimation problems.

The contributions of this article can be summarized thus:

- To the authors’ knowledge, this is the first work that explores the spectrum utilization via analysis of REM for channels occupied by active communicating UAVs, through measurements collected by a UAV-mounted SDR sensor. A description of the experiment performed in a real-world outdoor environment (a flight model club) is given, as well as graphical and numerical representations of the analyzed metrics (received signal mean power level, average difference of the mean power level, percentage of meaningful correlations).
- Exploration of the significance in temporal (2D space), spatial (3D space), and frequency domains of the received signal level variations. This analysis provides insight for the influence of the UAVs’ altitude and multipath reflectors on the REM, and identifies the RoIs in the particular environment. In this way, it serves as a basis for further research of reducing the amount of measurements and spectrum occupancy prediction for UAV-based communication coexistence in the ISM bands.

The rest of this paper is organized as follows. A review of the relevant literature to emphasize the findings of this work is provided in Section 2. The equipment used for the experiment’s execution and for the measurement data collection is described in Section 3. Section 4 explains the experimental setup. Analysis of the REM and its features in the temporal, spatial, and frequency domains are provided in Section 5. On this basis, Section 6 gives directions for future work, and Section 7 concludes this article.

## 2. State of the Art

Recent works on UAV-based collection of measurements for the construction and estimation of REMs in both 2D and 3D space are hereby reviewed. Due to the complexity and equipment costs of implementing real-time measurement campaigns via highly mobile UAVs, the majority of the available literature is based on simulation-driven experiments, primarily via RT but also statistical path loss (PL) models. Many of these works [21,23,24,29,31] construct REMs (also referred to channel knowledge maps, or CKMs in recent publications such as [25,33]) in a 3D space, even though if the research focus is on providing UAV-based measurements methodology or REM estimation from limited number of measurement samples [17,18,20,25,26,32], usually generalized 2D maps are presented. In addition, as explored by [24,25,26,33], a dynamic REM database can be used in UAV-powered communication scenarios to provide substantial throughput gains for cellular heterogeneous and other wireless networks. A summary of the reviewed works that includes their applications, goals, and technical details of the conducted experiments is given in Table 1.

The main research traits of the reviewed publications, as well as the relevant advantages of this work, are as follows:

- UAV-based measurements are often used to map a cellular network’s coverage. Thus, both 3D assessment of the signal quality and network coverage planning are provided for more precise decision-making in UAV–ground and UAV–UAV communications, considering the spectrum utilization fluctuations in time and space [6]. In this work, an REM in 3D space is constructed, and the significance of the received signal level dynamics in the temporal, spatial (in 2D and 3D space, respectively), and frequency domains is determined.
- Limited work has been carried out for REM construction from measurements performed via a UAV-based sensor due to the equipment costs and the difficulty of the experimental setup’s execution. The practical considerations (such as computational complexity and SDR capabilities) for constructing REMs from wideband signals are rarely made in the relevant literature, as its focus is usually on REM estimation and interpolation methods that are often agnostic toward the carrier frequency and bandwidth. They are, nevertheless, important parameters in practical studies and prototyping for UAV-based communications. This paper presents real-time experiments conducted by a UAV-mounted sensor that collects spectrum occupancy data for multiple active UAVs in an outdoor environment, and details the practical implementation of the measurement procedure. The goal of this work is to describe the spectrum utilization (through the variations of the received signal) of an ISM band in the condition of densely deployed aerial communication nodes.
- Due to the impracticality (UAV short flight time and physical unfeasibility of covering a very large volume of space in an urban environment [3,22,23]) of constructing a 3D REM using a complete set of measurements, it is usually obtained from a limited number of samples that represent only certain areas of the volume, or the RoI. Estimating the trajectory that covers the RoI is, therefore, an important field of research. This work analyzes the correlation in the temporal and frequency domains as well as the differences in signal level fluctuations, thus providing a base for further research in reducing the amount of measurements and spectrum occupancy prediction for UAV-based communication coexistence in the ISM bands.

## 3. UAVs and Measurement Equipment

**Figure 2:**
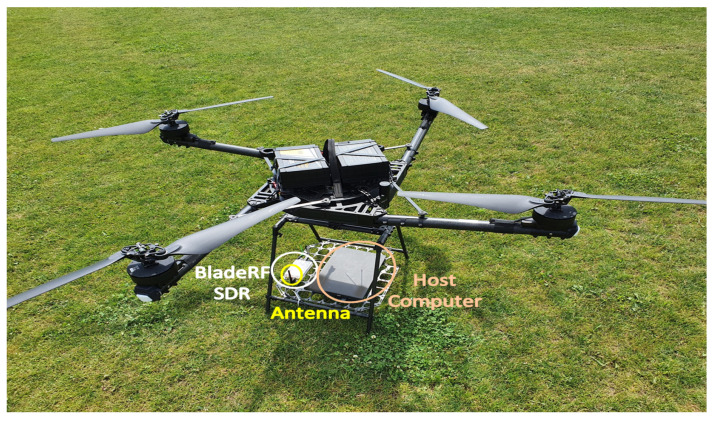
Freefly ALTA X (this is the **sensor UAV** that carries the SDR, the omnidirectional antenna, and the host computer, while the other three are the **sensed UAVs**).

**Figure 3:**
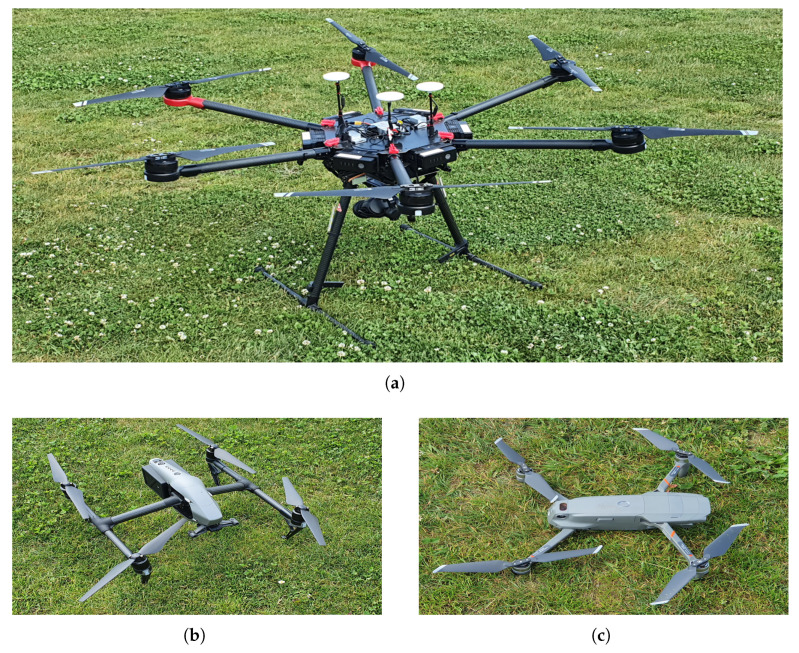
Sensed UAV models: (**a**) DJI Matrice 600 Pro. (**b**) DJI Inspire 2. (**c**) DJI Mavic 2 Enterprise Dual.

In the planning and execution of this experiment, the challenges for operating UAV-based measurement campaigns, presented in [34], are considered, and in this, as well as subsequent sections, the frequency, temporal, and spatial aspects of the implementation are denoted. This section describes the UAVs (which, together with their corresponding RCs, constitute the umanned aerial systems or UASs) and the measurement equipment that are employed for the execution of the experiment described in this work. Thus, in this section, as it is related to the planning of the experiment, most of the challenges are of the spatial aspects. There are three UAVs (referred to as “sensed UAVs”) that fly in a constellation around another UAV (a “sensor UAV”), which, on the other hand, carries a laptop and a lightweight SDR. The three sensed UAVs perform their flight in a constellation, playing the role of users of the spectrum, while the purpose of the study is to evaluate the spectrum occupancy and construct the REM in 3D. The sensor UAV follows a separate flight trajectory (at an altitude 10 m lower than that of the constellation of sensed UAVs), during which the SDR mounted on it records the signals at a frequency band utilized by the sensed UAVs. In this way, the sensor UAVs is recording the signals of the sensed UAVs, i.e., the experimental setup illustrated in Figure 1, is implemented. Then, the obtained RF data are used for an offline analysis of the spectrum occupancy characterization and 3D REM construction. It should be noted that this scenario is concerned only with characterizing the spectrum occupancy, and so distinguishing between the signals of the specific sensed UAVs is not considered. A brief description of the UAVs’ capabilities is provided in Table 2, and they are illustrated in Figure 2 and Figure 3. The UAVs employed for these experiments are the Freefly Systems (Woodinville, WA, USA) ALTA X, and the DJI (Shenzhen, Guangdong, China) Matrice 600 Pro, Inspire 2, and Mavic 2 Enterprise Dual.

**Figure 4:**
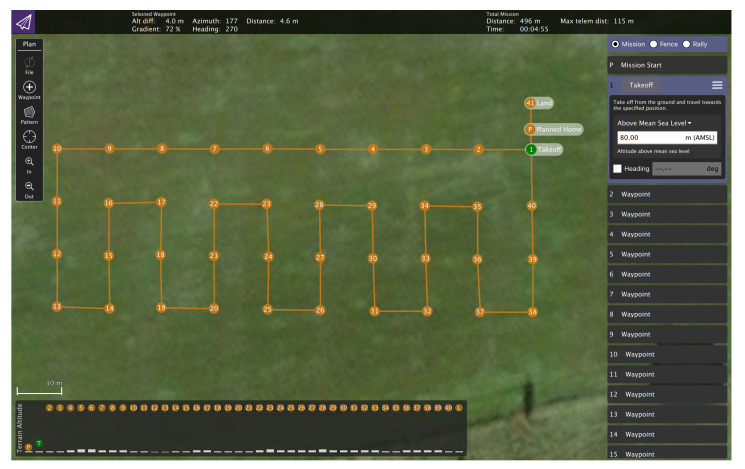
QGround control waypoint trajectory for the sensor UAV.

The radio controllers operating at various frequency bands, used for telemetry, command and control, and video reception purposes, that are utilized are the following: Futaba T14SG (controls the Freefly ALTA X), DJI Matrice 600 Pro Radio Controller, DJI Inspire 2 Remote Controller, and DJI Mavic 2 Enterprise Dual Smart Controller. For waypoint trajectory planning and execution of sensor UAV, missions are created in QGroundControl and uploaded before launch. Figure 4 shows a snapshot of the waypoint trajectory (including the points of takeoff, landing, and the 40 points which the sensor UAV covers during its flight) planned in QGroundControl.

Then, the capabilities (representing the frequency and temporal aspects) of the hardware used for recording the RF data are considered as follows. The laptop (weight of 1.3 kg, equipped with Core i3 5010U processor, 4 GB random access memory, solid state drive with speed of up to 6 GB/s, operating on Ubuntu) operates the SDR via a universal serial bus (USB) 3.0 connection, both being mounted on the sensor UAV as shown in Figure 2. The Nuand (New York, NY, USA) BladeRF 2.0 micro SD is utilized for this experiment due to its accessible price, small size, and satisfactory processing capabilities [39]. It is applicable for UAV-based measurements due to its compact dimensions (11 cm × 7.4 cm × 2.5 cm), weight of about 200 g, and the capability to process wideband signals of up to 56 MHz in the range between 47 MHz and 6 GHz owing to the notable AD9361 transceiver frontend [40]. A crucial feature is the USB 3.0 connection that allows the recording and processing of bit rates up to 5 GB/s (or bandwidth of about 78 MHz at a rate of 64 b/sample) [41]. Nevertheless, preliminary empirical tests are necessary to examine the maximum bandwidth that can be recorded on the host computer by the bladeRF. To cover the UAVs’ control bands (2.4 GHz), the SDR is operated via GNU Radio [42] and a standard omnidirectional ISM-band antenna with 2 dBi gain and a compact length of about 12 cm (see Figure 2).

## 4. Experimental Setup and Measurement Methodology

The experimental setup (relates to the temporal, frequency, and spatial aspects of the experiment’s implementation) is implemented using the considerations presented in [34], and is composed of the three sensed UAVs and the sensor UAV with the SDR and host computer mounted on it, described in Section 3. The experiment is performed for one day at the Silkeborg El & Svæv field for model and UAV flights, located in the area of Silkeborg, Denmark.

**Figure 5:**
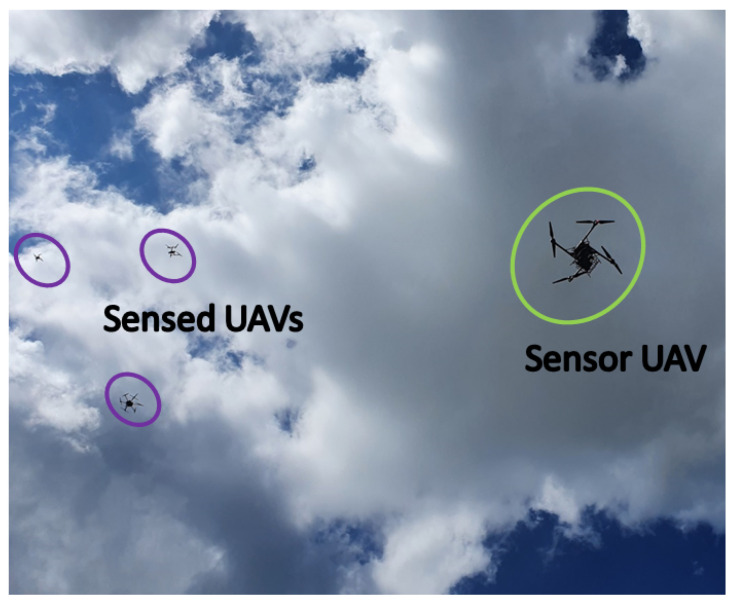
Sensor UAV (in green) and sensed UAVs (in violet) in flight.

**Figure 6:**
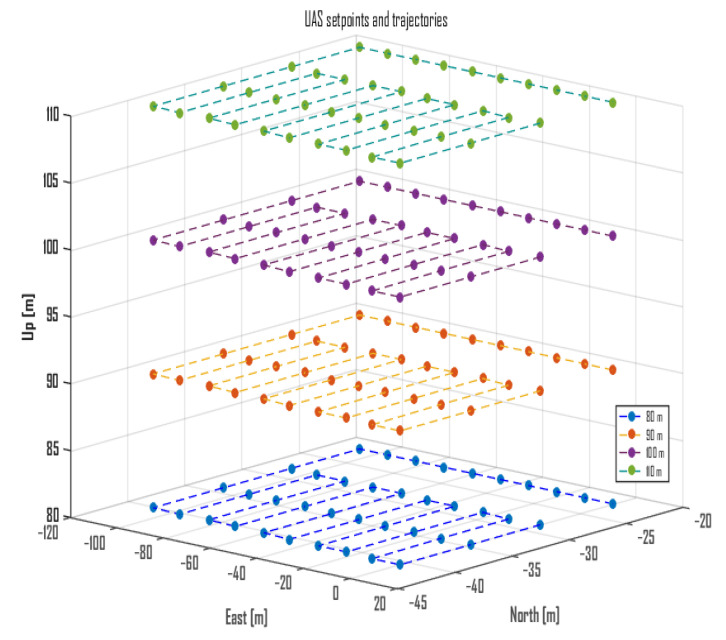
Trajectory of the sensor UAV.

The sensed UAVs follow a predetermined realistic flight trajectory that forms a constellation and is designed to be safe from possible collisions. They fly at an altitude approximately 10 m higher than the trajectory followed by the sensor UAV (Figure 1 and Figure 5). As the constellation of sensed UAVs performs its flight, the data exchange transmissions with their RCs are recorded at a band of 20 MHz width by the sensor UAV. Its operation is described as follows. Four altitudes (denoted by $H_{S}$) are defined—80, 90, 100, 110 m above sea level (10, 20, 30, and 40 m above the ground, respectively), at which the sensor UAV is to fly, while its trajectory is designed to follow 40 points, as shown in Figure 6. These points (coordinates or locations) cover an area that allows for the sensed UAVs to fly around in a constellation—it encompasses 3990 m${}^{2}$ (105 m × 38 m). The sensor UAV hovers at the coordinates of each point for 5 s before moving to the next one (the movement takes around 4.7 s); thus, the flight trajectory’s execution is estimated to take about 380 s (considering the inconsistency of movement and hovering periods caused by the wind). The trajectory of the sensor UAV is defined with consideration of the area over which such flights are permissible and feasible, and based on it, the trajectories of the constellation of sensed UAVs are defined.

Before the start of the sensor UAV’s flight, a Python script records the 20 MHz bandwidth in a single file on the computer for 500 s (including the periods of reaching the altitude and executing the flight trajectory). Afterward, the sensed UAVs are set to start the execution of their trajectories together with the sensor UAV. This process is repeated four times for each altitude.

**Figure 7:**
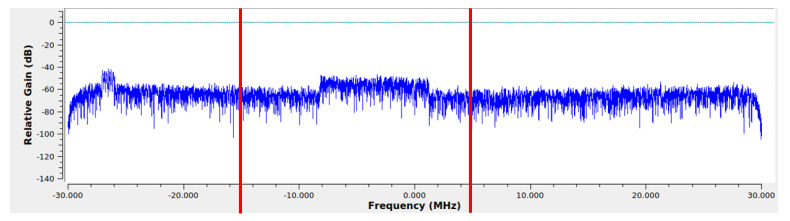
The GNU Radio’s spectrum graphic (range 2.4–2.46 GHz). The red lines outline the channel’s width.

To determine the frequency band that will be measured, the sensed UAVs are set into a preliminary flight trajectory (while the sensor UAV is offline) and, through a manual search (using the GNU Radio’s spectrum graphic, Figure 7), a channel that has significant and frequent emissions is chosen. In this case, that channel is the one with central frequency of 2.427 GHz (20 MHz bandwidth). It should be noted that, in this work, the focus is on the evaluation of spectrum occupancy, and not simply on the channel between a transmitter and receiver. Thus, the particular source(s) (among the three sensed UAVs) of the signal(s) in the analyzed band at each moment in time is not considered, i.e., the analysis regards the presence or absence of signal emissions (the only transmissions can be produced by the sensed UAVs within the region of space), and not the detection of the specific sources or their number. Interference between the three sensed UAVs in one and the same band is unlikely as their communication signals do not necessarily employ the entire 20 MHz band. Meanwhile, preliminary measurements in the region where the experiment is performed show that there are no interfering ground communication nodes in the 2.4 GHz spectrum.

## 5. Data Analysis and REMs

The obtained measurement data are processed for analysis in time, frequency, and spatial domains in the following manner. A separate file with duration of 500 s, containing RF complex in-phase/quadrature (IQ) data, is generated from the SDR for each of the four altitudes, $H_{S}=\left\{80,90,100,110\right\}$ m, on which the measurements were performed. Once they are recorded, each file is processed through a filter that reduces the direct current (DC) component that is characterized by a large spike in the center frequency, usually produced by most SDRs. This filter is implemented as a “DC Blocker” block in GNU Radio with delay line of length 1024 for a more thorough DC component suppression [43].

Afterward, using the sensor UAV’s control logs, the starting and ending times of the period during which the UAV covered its programmed trajectory of 40 points (Figure 6) is estimated. Consequently, the chunk of samples that corresponds to this period (of around 380 s) for each altitude is considered for processing, while the rest of the data are discarded. These samples are then processed in the temporal, spatial, and frequency domains as follows.

### 5.1. Analysis in the Time Domain

**Figure 8:**
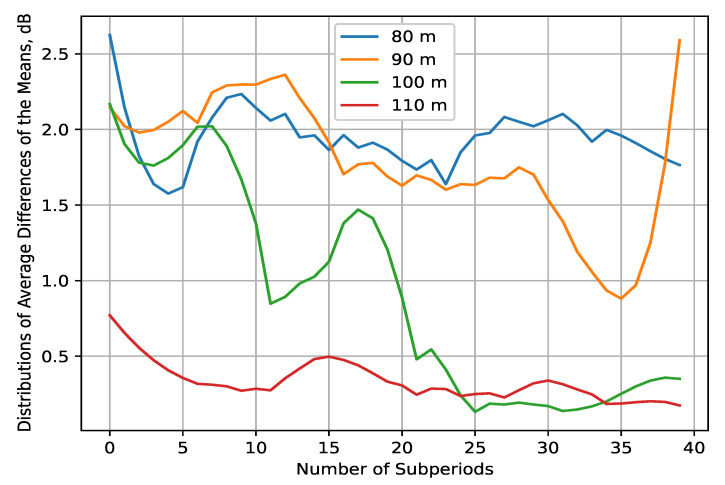
Distributions of average differences in time domain between the means of the main processing dataset.

The data analysis in the time domain is hereby performed by first taking the IQ samples’ mean for several temporal granularity values (1 ms/5 ms/10 ms/50 ms/1 s) for each of the four measurement files (corresponding to the four altitudes at which the measurements were made). As a result, the *main processing dataset* is formed. The temporal granularity for which the analysis is made is determined empirically depending on the meaningfulness provided by the results (which is usually characterized by the intensity of their fluctuations). Thus, granularity of 10 ms is chosen as it is to be expected that the received signal level variations will be meaningful for time delays of such proportions in the general case of UAV air-to-air (A2A) communications channel [44]. First, the differences between every two samples in the main processing dataset are found, and then their mean is computed for each of the 40 points at which the UAV hovers. In this way, the distributions of average differences in the received signal’s means can be obtained (Figure 8). These distributions illustrate the following tendencies. Firstly, it is notable that the dynamics of the signal’s mean power decline in intensity with the altitude, as the differences at $H_{S}=\left\{80,90\right\}$ m are significant (i.e., over 1 dB) for almost the whole measurement period, while that is true for about half of the measurements at $H_{S}=100$ m. As for the distribution of differences at $H_{S}=110$ m, there are no meaningful changes in the means. The differences are on average, close to or over 2 dB at $H_{S}=\left\{80,90\right\}$ m, thus showing the wide-range variations in the signal propagation for these lower altitudes. Consequently, it is seen that from the much lower intensity of the received mean power change with time at $H_{S}=100$ m, the propagation conditions differ substantially at this altitude. These observations are explained by the main characteristics of the A2A channel for UAV communications [45,46,47]:

- As the altitude increases, the propagation conditions can be approximated by the free space path loss; thus, they are favorable in the 2.4 GHz frequency range. The path loss exponent also declines exponentially.
- Tall objects that create multipath components in the received signal, as well as ground reflections, tend to become negligible at a certain altitude (in this case, for $H_{S}\geq 100$ m).
- The differences in velocity and direction between the sensed UAVs and the sensor UAV, especially at lower altitudes ($H_{S}<100$ m), contribute to a significant signal variation. In addition, the same effect is caused by the UAVs’ airframe shadowing, particularly in the sensor UAV due to its aerial structure obstructing the line of sight between the transmitting UAVs and the SDR.

**Figure 9:**
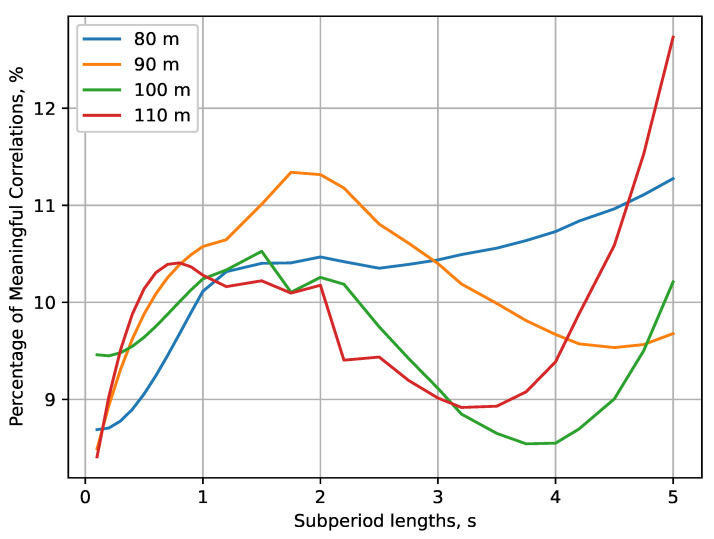
Distributions of the percentage of meaningful correlations of the main processing dataset in time domain.

To further this analysis, the correlation between the means for subperiods $T_{C}$ in the interval $\left[0.1;5\right]$ s is estimated throughout the whole measurement time at all four altitudes for each subperiod. The Spearman correlation coefficient is used as the received complex signal is not normally distributed [48]. If the *p*-value of the Spearman test is lower than 0.05, the correlation between two sets with length of one subperiod is considered to be meaningful. The distributions of the percentage of meaningful correlations for the interval of subperiods is shown in Figure 9. It illustrates that the percentage varies in a small range between 8.5 and 12.7% and, thus, it does not provide much information beyond corroborating with the current findings. For subperiod lengths close to 5 s (correlation between the samples corresponding to the points over which the sensor UAV hovers without moving), the most significant correlations are shown to be on the lowest, $H_{S}=80$ m, and highest, $H_{S}=110$ m, altitudes. As shown in Figure 8, the average differences in these two cases show small variations (of small and high proportions, respectively) that can be attributed to the correlations’ percentage (both being over 10%). On the other hand, the results for the other two altitudes exhibit stronger fluctuations and, concurrently, smaller percentage of correlations.

### 5.2. Analysis in the Spatial Domain

**Figure 10:**
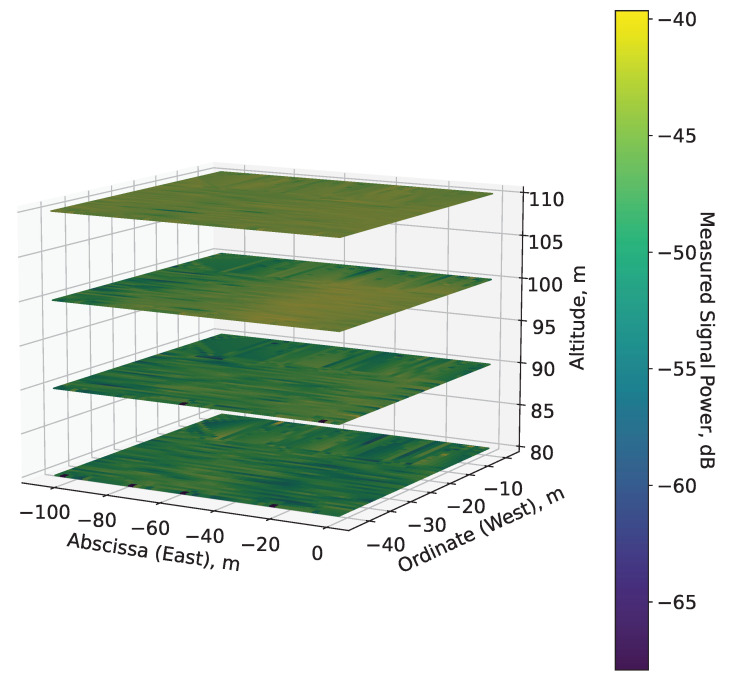
3D REM.

In this subsection, the distribution of the main processing dataset in space is analyzed. Firstly, a 3D REM is constructed by applying standard linear interpolation to the dataset for each altitude (Figure 10). It encompasses 3990 m${}^{2}$, including the abscissa axis $X:x\in \left[-105;0\right]$ m (corresponding to eastern direction) and the ordinate axis $Y:y\in \left[-42;-4\right]$ m (corresponding to western direction). To facilitate the analysis, the X axis can be understood as rows, while the Y axis as columns of the interpolated 2D data matrix I. At temporal granularity of 10 ms, the surface plot on each plane is composed of approximately 38,000 samples (the interpolation resolution is thus equal to $\left\lfloor \sqrt{38,000}\right\rfloor$) for all four altitudes, thus illustrating the distribution of the mean received signal power in 3D space and its fluctuations in height. In this way, the four 2D distributions (one on each altitude) form an REM in 3D space. Then, the analysis of the collected data accounts for the different altitudes, which illustrates the variations in the UAV A2A channel in 3D. The most obvious feature is the significant decline in the power level change with higher altitude, leading to consistent strong signal reception at $H_{S}\geq 90$ m. The rate of variation is most significant at the lowest altitude $H_{S}=80$ m, while it decreases to an extent in the higher elevations. For all four altitudes, there are notable tendencies of similar signal power levels along the ordinate axis for $x\in \left[-105;0\right]$ m and $y\in \left[-15;-4\right]$ m. Likewise, there are multiple instances of correlated measurements along the abscissa axis for $x\in \left[-105;0\right]$ m and $y\in \left[-40;-15\right)$ m. The received signal power has a high magnitude almost invariably in the range of $x\in \left[-40;0\right]$ m; $y\in \left[-42;-15\right)$ m at $H_{S}=100$ m, as well as for the whole measurement plane at $H_{S}=110$ m. At that latter altitude, notable signal variations are present in only a negligible area. The measured signal level is high with negligible variations at this height, as the sensed UAVs are flying at an altitude just 10 m higher than that of the sensor UAV; thus, the distance between them is usually in the span of up to several dozens of meters. In addition, as observed in Figure 8, the average differences of the means of the received signal power are very small (less than 0.5 dB). 

**Figure 11:**
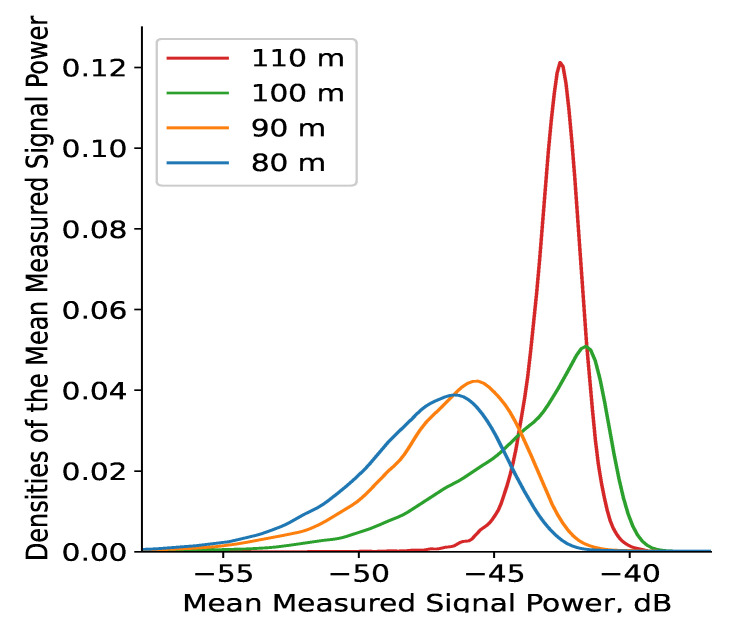
Densities of the mean measured signal power in spatial domain.

The distribution densities (Figure 11) provide a more analytical expression of the spatial dynamics of the same mean measured signal power illustrated graphically by the 3D REM (Figure 10). These densities show that the mean signal variance is very significant in the span of 15 dB (between −58 and −43 dB) for $H_{S}<100$ m. The mean signal powers for $H_{S}=80$ and 90 m follow very similar distributions, with the vast majority of their values being between −50 and −43 dB, which corresponds to the many instances of rapid change in the REMs that are in the same interval (illustrated in brighter colors, according to the color map of Figure 10). There are, though fewer in number, some instances of weak signal power (<−50 dB), which reflect the notable precedents of such levels in the REMs at the low altitudes. These dynamics corroborate with the significant changes in the measured signal power illustrated by the REMs. The distributions for the two highest altitudes have very different and distinct densities, even though for both of them, the majority of measured signal powers are much higher (>−43 dB) than those for the lower altitudes. In addition, the spread of the densities for $H_{S}$ of 100 and 110 m is much smaller, which corresponds to the notably smaller variance in the REMs. In accordance with the REM for $H_{S}=100$ m, in Figure 10, there is a meaningful variance for signal levels in the interval between −55 and −45 dB for certain regions of the measurement space. This variance becomes insignificant at the highest altitude, as it is only within the span of about 3 dB, thus establishing the stable high-level reception for the entirety of the measurement plane.

**Figure 12:**
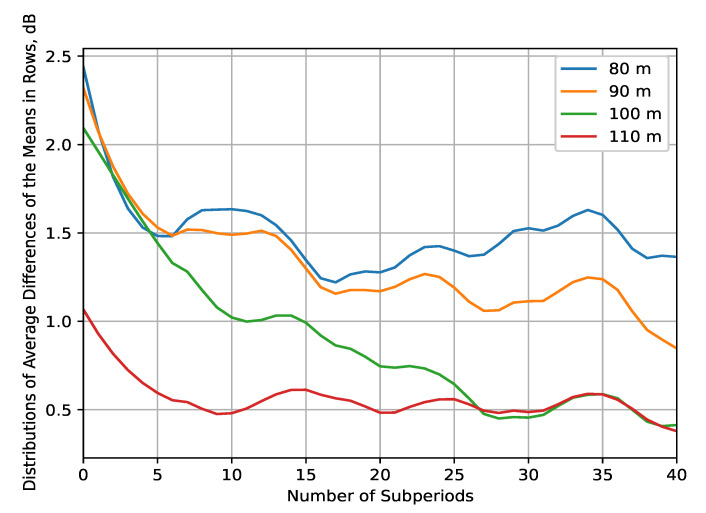
Distributions of average differences in rows between the means of the interpolated 2D data for each altitude.

**Figure 13:**
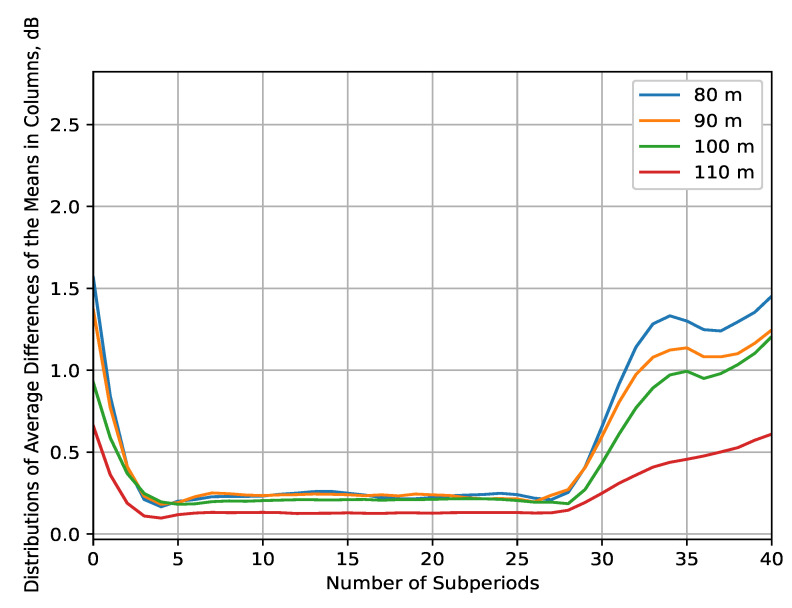
Distributions of average differences in columns between the means of the interpolated 2D data for each altitude.

A closer look at the tendencies in the variations of mean measured signal power, illustrated in Figure 10, is provided by estimating the distributions of average differences of the means per rows (Figure 12) and per columns (Figure 13). They are constructed in a similar manner to those in Figure 8, but so that the interpolated data are processed by rows and by columns, respectively, with a subperiod that is 40 times smaller than the interpolation resolution of the grid. The purpose is to analyze the received signal fluctuations in the *x*-axis (in rows) and in the *y*-axis (in columns) to determine the most significant regions of space (i.e., where the received signal variance is the greatest). These regions are the ones that require the most measurement data and prediction algorithms, being the RoIs. The results in Figure 12 have the following features. There are large average differences in the means (>1.5 dB) in the regions close to the starting points of every row for up to the fifth subperiod (corresponding to $x\in \left[-105;-90\right]$ m; $y\in \left[-42;-4\right]$ m, or around 14% of the planes’ surface, at $H_{S}=\left\{80,90,100\right\}$ m in the REM in Figure 10). Further confirming the results of Figure 8, there is a considerable variation in the differences for the whole measurement range at $H_{S}=\left\{80,90\right\}$ m, and for almost 40% of it at altitude of 100 m, which corresponds to $x\in \left[-105;0\right]$ m; $y\in \left[-42;-4\right]$ m and $x\in \left[-105;-65\right]$ m; $y\in \left[-42;-4\right]$ m.

By performing the same processing on the columns of the interpolated data matrix, it is illustrated in Figure 13 that the changes in the signal levels are not significant for the majority of the measurement area. Nevertheless, there is a notable increase in the average differences for the last 10 subperiods at $H_{S}=\left\{80,90,100\right\}$ m (corresponding $x\in \left[-105;0\right]$ m; $y\in \left[-13;-4\right]$ m, or around 24% of the planes’ surface), which is also visible in Figure 10. The fluctuations are, again, shown to grow weaker as the altitude increases.

### 5.3. Analysis in the Frequency Domain

To extend the scope of the spectrum occupancy characterization, an analysis in the frequency domain is also made. The 20 MHz bandwidth is separated into four chunks (subbands) of 5 MHz via an elliptical filter of 30th order, ripple coefficient of 0.5 dB, and minimum attenuation in the stop band of 60 dB (these parameters are determined empirically). For a temporal granularity of 10 ms, the mean for each subband is estimated, resulting in around 38,000 samples per chunk in the *subband dataset*. Then, in a similar fashion to the analysis in Section 5.1, the Spearman correlation is computed between every two batches of samples (their length is 500 samples, corresponding to 5 s) that correspond to the locations over which the sensor UAV hovers at a specific location within each subband dataset for all four altitudes. The resulting percentages of meaningful correlations are given in Table 3.

There is only a small amount of substantial correlation within the subbands at $H_{S}=\left\{80,90,100\right\}$ m, thus showing that in the case of a highly dynamic propagation environment, decreasing the observed bandwidth does not influence the RF data temporal trends. At $H_{S}=110$ m, there is a notable increase (up to 20%) of considerable correlations in Subband 1, which shows that it contributes the most to the nearly uniform distribution of the received signal’s means at that altitude, as discovered in Section 5.1 and Section 5.2. Therefore, in the case of highly correlated samples, the impact of particular subbands’ utilization is significant. Nevertheless, its variance in time is not necessarily smaller than that of the overall band. At the two lowest altitudes, the percentages of meaningful correlations in Subband 1 are close to those of the whole band (Figure 9), with some slight decline for $H_{S}=100$ m, followed by a significant increase for $H_{S}=110$ m. This pattern shows an increase in stationarity with altitude, which is, however, not relevant to the other subbands (for which the change in altitude is somewhat sporadic), as there is no clear dependency between their percentage of correlations and the change in height. Nevertheless, these observations are generally in accordance with the established high degree of variation observed for the UAV A2A channel in the frequency domain for bandwidths over 1 MHz and altitudes over 100 m [47].

## 6. Discussion and Directions for Future Work

The results of this experiment reveal the following features of the REM for UAV A2A communications:

- At low altitudes ($H_{S}<100$ m), the velocity difference between the sensor UAV and sensed UAVs, and the reflection from the ground and nearby tall objects have a significant effect on reception. The received signal’s fluctuations are non-negligible, and vary in the span of at least several dB, as illustrated by the REM (Figure 10). For this reason, it is unlikely that a reliable RoI (that does not include the whole measurement area) may be defined at such elevation levels. Nevertheless, this observation is the reason to suggest high computationally efficient low-altitude spectrum sensing. Either probabilistic [49] or machine-learning-based [50] algorithms for real-time spectrum sensing can be utilized to process the RF samples in less than 10 ms for accurate occupancy characterization. Its application in low-altitude UAVs [51] is, thus, an interesting research direction.
- The fluctuations’ intensity, as shown in Figure 9, Figure 12 and Figure 13, decreases dramatically with altitude (in this case, for $H_{S}\geq 100$ m) due to the much weaker influence of ground reflection, whereas for higher altitudes, notable signal variations are present in only negligible portions of the plane (as the average difference of the means is around 0.5 dB for $H_{S}=110$ m). Then, the RoIs can be identified much more reliably as the areas in the REM at each elevation level in which the difference between the signal means is meaningfully high (>1 dB). For the rest of the REM, compressed sensing and limited measurements can be used for accurate spectrum reconstruction. Then, the REM database can be utilized instead of real-time spectrum sensing to characterize the resource availability.
- Signal variance has different distributions depending on whether the sensor UAV moves in the *x*- or *y*-axis (Figure 12 and Figure 13). For $H_{S}\leq 100$ m, the fluctuations in rows (Figure 12) are observable just as in the time domain distributions (Figure 8); however, they allow for a more precise determination of the RoIs that corroborates with the visual illustrations present in the REM (Figure 10). Much less information is provided by the distribution of average means in the ordinate axis (Figure 13). The RoIs, as defined by their coordinates in Section 5.2, constitute nearly the whole surface for $H_{S}=\left\{80,90\right\}$ m, but there is evidence that it may be decreased, as the most intensive variations comprise about 38% of it. The same can be said for the results at $H_{S}=100$ m, even though the whole RoI is about 75%. Due to the very favorable propagation conditions, the RoI at $H_{S}=100$ m is only marginal. These observations show that the sensor UAV’s direction of flight affects the signal variance, so that movement in a certain axis (the abscissa in this case) yields more meaningful measurement results, thus providing insight into the important field of path-planning for UAV communications [52].
- The frequency domain analysis shows that for weakly correlated signals, the mean power in the subbands does not significantly contribute to the information about spectrum utilization. Nevertheless, in the case of strong reception, there is a high correlation in particular subbands that reveals the nonuniformity of the sensed UAV communication signals in the frequency domain. They do not occupy the whole ISM channel, as is usually the case with other license-free wireless standards, which provides the opportunity for further studies to explore the potential for increased spectrum utilization in bands of 5 MHz width. This consideration may benefit the investigation of applications such as transmitting source identification through modulation classification, or duty cycle estimation [53].

## 7. Conclusions

This work presents a sophisticated real-world experiment for the study of spectrum utilization in the A2A channel for UAV communications. An SDR-enabled sensor UAV performs the measurements of the sensed UAVs’ signals, as they fly in a constellation around it. Using the gathered RF data, a volumetric (in 3D space) REM is constructed, and a foundational analysis in the time, spatial, and frequency domains is made. These results illustrate some important characteristics of the A2A channel, and the received signal’s variations provide insight for further work into algorithms for REM estimation based on limited measurements, REM prediction, spectrum sensing, and transmitter source identification. The collected data can also be used for comparison and verification of such methods.

## Figures and Tables

**Table 1:** Summary of relevant works in the field of UAV-based measurements for REM construction and estimation.

Reference	Application	Contribution	Realization of Experiments	Transmitter Sources	Carrier Frequency	Bandwidth	Notes
[17]	Cellular coverage mapping	Methodology for UAV-based measurements and REM construction	Real-time measurement collection via UAV-mounted UEs	Single stationary LTE BS	800 MHz	20 MHz	Custom drone and measurement hardware
[18]	mmWave measurement system	UAV-based REM measurements methodology; path loss and radiation patterns estimation	Real-time measurement collection via a lightweight spectrum analyzer	Single stationary mmWave transmitter	28 GHz	≈1 GHz	Custom drone and measurement hardware
[19]	REM construction from limited measurements	REM estimation via statistical signal inference	Simulation via RT and statistical modeling	Single stationary transmitter	100 MHz	5 MHz	-
[20]	UAV flight path optimization	REM estimation via deep autoencoders combined with a Bayesian estimator	Simulation via RT and statistical modeling	Multiple stationary transmitters	2.4 GHz	5 MHz	-
[21]	Cellular coverage mapping; UAV flight path optimization	3D REM estimation from limited UAV measurements	Simulation via statistical modeling	Multiple stationary transmitters	Not applicable (N/A)	N/A	Number of measurement locations determined
[22]	UAV flight path optimization	3D REM interpolation from limited measurements	Simulation via statistical modeling	Multiple stationary transmitters	100 MHz	200 kHz	-
[23]	UAV flight path optimization	3D REM interpolation from limited measurements	Simulation via statistical modeling	Multiple stationary transmitters	100 MHz	200 kHz	-
[24]	Cellular coverage mapping	UAV-based REM measurements for 5G network performance assessment	Real-time measurement collection via a UAV-mounted UE	Single stationary 5G BS	3.5 GHz	100 MHz	Custom drone, measurement software, and hardware
[25]	UAV flight path optimization for numerous UAV-BS connections	REM interpolation from limited measurements; throughput maximization for UAV communications	Simulation via RT	Multiple stationary cellular BSs	N/A	N/A	-
[26]	UAV flight path optimization; prediction of BS coverage outage regions;	Minimization of flight time and outage duration via DRL	Simulation via statistical modeling	Multiple stationary cellular BSs	N/A	N/A	-
[29]	TV channels coverage mapping	3D REM interpolation	Real-time measurement collection via a stationary SDR	Single stationary TV transmitter	470–590 MHz	25 MHz	Sensing performed via USRP SDR
[31]	REM construction from limited measurements	3D REM estimation via orthogonal matching pursuit	Simulation via statistical modeling	Multiple stationary transmitters	N/A	200 kHz	-
[32]	REM construction from limited measurements	REM estimation via deep autoencoders	Simulation via RT and statistical modeling	Multiple stationary transmitters	2.4 GHz	5 MHz	-
**This work**	Spectrum occupancy characterization for UAV communications	REM-based analysis of the ISM band in the temporal, spatial, and frequency domains	Real-time measurement collection via UAV-mounted SDR	Multiple airborne UAV nodes	2.4 GHz	20 MHz	Sensing performed via BladeRF SDR

**Table 2:** Summary of the UAVs’ operational capabilities.

UAV Model	Dimensions	Radio Controller	Operating Frequency	Maximum Flight Time
Freefly ALTA X (sensor UAV) [35]	Diameter (unfolded)—2273 mm/Height—387 mm	Futaba T14SG	2.4 GHz	≈15 min
DJI Matrice 600 Pro [36]	1668 mm × 1518 mm (unfolded)/Height—727 mm	Dual remote control with HDMI output	5.725–5.825 GHz; 2.400–2.483 GHz	≈38 min
DJI Inspire 2 [37]	Diameter—605 mm	Dual remote control with HDMI output	2.400–2.483 GHz; 5.725–5.825 GHz	≈27 min
DJI Mavic 2 Enterprise Dual [38]	322 mm × 242 mm (unfolded)/Height—84 mm	Smart Controller with OcuSync 2.0	2.400–2.4835 GHz; 5.150–5.250 GHz; 5.725–5.850 GHz	≈27 min

**Table 3:** Percentages of meaningful correlations within each subband of the subband dataset.

	Subband 1: 0–5 MHz	Subband 2: 5–10 MHz	Subband 3: 10–15 MHz	Subband 4: 15–20 MHz
**Altitude 80 m**	10.53%	10.53%	5.26%	2.63%
**Altitude 90 m**	10.53%	0%	2.63%	5.26%
**Altitude 100 m**	7.69%	0%	7.69%	2.56%
**Altitude 110 m**	20.51%	7.69%	2.56%	10.26%

## Data Availability

The data for replication of the results are available from the authors upon request.

## Abbreviations

The following abbreviations are used in this manuscript:

2D	Two-dimensional
3D	Three-dimensional
5G	Fifth Generation
A2A	Air-to-air
BS	Base station
CKM	Channel knowledge map
CR	Cognitive radio
DSA	Dynamic spectrum access
ISM	Industrial, Scientific, and Medical
IQ	In-phase/quadrature
KPI	Key performance indicator
PL	Path loss
QoS	Quality of service
RC	Remote control
REM	Radio environment map
RF	Radio frequency
RoI	Region of interest
RT	Ray tracing
RSS	Radio signal strength
SDR	Software-defined radio
TV	Television
UAV	Unmanned aerial vehicle
UAS	Unmanned aerial system
